# Levels of α7 integrin and laminin-α2 are increased following prednisone treatment in the *mdx* mouse and GRMD dog models of Duchenne muscular dystrophy

**DOI:** 10.1242/dmm.012211

**Published:** 2013-07-11

**Authors:** Ryan D. Wuebbles, Apurva Sarathy, Joe N. Kornegay, Dean J. Burkin

**Affiliations:** 1 Department of Pharmacology, University of Nevada School of Medicine, Reno, NV 89557, USA; 2 Department of Veterinary Integrative Biosciences, Texas A&M University, College Station, TX 77843-4458, USA

## Abstract

Duchenne muscular dystrophy (DMD) is a fatal neuromuscular disease for which there is no cure and limited treatment options. Prednisone is currently the first line treatment option for DMD and studies have demonstrated that it improves muscle strength. Although prednisone has been used for the treatment of DMD for decades, the mechanism of action of this drug remains unclear. Recent studies have shown that the α7β1 integrin is a major modifier of disease progression in mouse models of DMD and is therefore a target for drug-based therapies. In this study we examined whether prednisone increased α7β1 integrin levels in *mdx* mouse and GRMD dog models and myogenic cells from humans with DMD. Our results show that prednisone promotes an increase in α7 integrin protein in cultured myogenic cells and in the muscle of *mdx* and GRMD animal models of DMD. The prednisone-mediated increase in α7 integrin was associated with increased laminin-α2 in prednisone-treated dystrophin-deficient muscle. Together, our results suggest that prednisone acts in part through increased merosin in the muscle basal lamina and through sarcolemmal stabilization of α7β1 integrin in dystrophin-deficient muscle. These results indicate that therapies that target an increase in muscle α7β1 integrin, its signaling pathways and/or laminin could be therapeutic in DMD.

## INTRODUCTION

Duchenne muscular dystrophy (DMD) is a lethal X-linked neuromuscular disease that affects 1 in 3500 boys. Clinical symptoms are first detected at 2–5 years of age and individuals with DMD often die from cardiac or respiratory failure by the second or third decade of life. DMD is caused by mutations in the dystrophin gene that lead to loss of the dystrophin protein (Monaco et al., 1986; Hoffman et al., 1987). Dystrophin and the associated protein complex link laminin in the extracellular matrix to the cell cytoskeleton and serves as a structural and signaling platform in muscle. Loss of dystrophin in the *mdx* mouse, golden retriever muscular dystrophy (GRMD) dog and DMD patients results in a fragile sarcolemma prone to contraction-induced muscle injury. Damaged muscle activates satellite cells to repair muscle damage, and rounds of muscle degeneration followed by regeneration occurs. Regeneration eventually fails and muscle is replaced with fibrotic and fatty tissue (Pichavant et al., 2011). There is currently no cure for DMD: however, improved medical care and treatment with corticosteroids, prednisone or deflazacort have improved muscle strength and longevity of patients (Fenichel et al., 1991; Angelini et al., 1994; Reitter, 1995; Merlini et al., 2003; Beenakker et al., 2005).

The *mdx* mouse model has provided valuable insights into the functional role of dystrophin in muscle. Although these mice exhibit muscle damage at the cellular level, outwardly they show little sign of muscle pathology (Bulfield et al., 1984). Short-term prednisone therapy in the *mdx* mouse has been shown to improve muscle strength (Sali et al., 2012). The GRMD dog model develops progressive and fatal muscle disease and exhibits pathophysiological disease features similar to DMD, including progressive loss of muscle function, muscle membrane fragility, cardiomyopathy and premature death (Cooper et al., 1988; Kornegay et al., 1988; Kornegay et al., 2012). Studies indicate that short-term treatment with prednisone has functional benefits in the GRMD model (Liu et al., 2004). Although corticosteroids are the current front line treatment for DMD and show short-term benefits in animal models of this disease, the mechanism(s) by which this drug improves clinical outcomes remains unknown.

α7β1 integrin is the predominant laminin-binding integrin in cardiac and skeletal muscle (Burkin and Kaufman, 1999). The α7β1 integrin protein is localized at neuromuscular and myotendinous junctions and extrajunctional sites in skeletal muscle (Martin et al., 1996; Burkin and Kaufman, 1999). In skeletal muscle, six isoforms of the α7 integrin chain are produced by developmentally regulated RNA splicing (Song et al., 1993). Mutations in the α7 integrin gene cause congenital myopathy in both humans and mice (Mayer et al., 1997; Hayashi et al., 1998; Flintoff-Dye et al., 2005). Enhanced transgenic expression of α7 integrin in the skeletal muscle of severely dystrophic mice improves muscle pathology and increases lifespan (Burkin et al., 2001; Burkin et al., 2005). Conversely, loss of α7 integrin in dystrophin-deficient *mdx* mice results in a more severe dystrophic phenotype and reduced viability, with mice dying prematurely by 4 weeks of age (Guo et al., 2006; Rooney et al., 2006). Together, these results support the idea that α7β1 integrin is a modifier of muscle disease progression in DMD and a target for drug-based therapies.

To investigate whether glucocorticoids act to increase α7β1 integrin in muscle, we examined levels of α7 integrin in myogenic cells from individuals with DMD as well as in the *mdx* mouse and GRMD dog animal models of DMD treated with prednisone. Our results show that treatment with prednisone promotes a dose-dependent increase in α7 integrin in mouse and DMD myogenic cells. In addition, we show the skeletal muscle of *mdx* mice and GRMD dogs treated with prednisone exhibit elevated levels of laminin-α2 and α7 integrin. Our results show for the first time that prednisone promotes an increase in the α7β1 integrin in muscle, which might contribute to the mechanism of action of this important therapeutic agent for DMD.

**TRANSLATIONAL IMPACT**

**Clinical issue**

Duchenne muscular dystrophy (DMD) is a fatal neuromuscular disease that affects 1 in 3500 newborn boys and is caused by mutations in the gene encoding dystrophin, a protein that supports muscle fiber strength. Loss of dystrophin results in reduced muscle cell adhesion to laminin in the basal lamina, which leads to progressive muscle damage. DMD is diagnosed at 3–5 years of age on the basis of failure to achieve movement-based milestones. Individuals with DMD are often confined to a wheelchair in their teens, require ventilator assistance to breathe and die in their second or third decade of life as a result of cardiopulmonary failure. There is currently no cure for DMD and treatment options are limited to glucocorticoid therapy, which has been shown to improve clinical outcomes including increased muscle strength. Although prednisone, a synthetic corticosteroid drug, has been used to treat DMD for decades, the molecular mechanisms that underlie the improvement in muscle strength are unknown.

**Results**

In this study the authors aimed to determine whether prednisone acts to increase levels of α7β1 integrin, an adhesion molecule previously shown to ameliorate muscle disease in mouse models of DMD. Using muscle cells cultured from mice or DMD patients, the authors show that prednisone acts to increase protein levels of α7 integrin in a dose-dependent manner. They also report that prednisone treatment in the well-established *mdx* mouse model of DMD and the GRMD canine model increases protein levels of α7β1 integrin, as well as those of laminin-α2, which makes up a component of the basement membrane. Finally, the authors show that GRMD dogs that have not been treated with prednisone exhibit reduced levels of laminin-α2 and α7 integrin proteins.

**Implications and future directions**

In this translational study the authors provide evidence that prednisone, the current front line treatment for DMD, acts in part to increase laminin-211/221 (composed of α2, β1 and γ1 chains) in the muscle basal lamina to stabilize protein levels of α7β1 integrin in skeletal muscle cells. These changes would lead to improvements in muscle fiber integrity in dystrophin-deficient muscle to slow the disease process. The results also suggest a shared mechanism for disease progression in GRMD dogs and humans, reinforcing the view that the canine model provides a useful tool for studies of the human disease. Despite the beneficial effects of prednisone, the improvement is temporary and, furthermore, therapy is complicated by the wide range of associated negative side effects. Future studies to identify drugs that specifically target an increase in laminin-α2 and/or α7β1 integrin in muscle are likely to improve clinical outcomes and have fewer negative side effects for DMD patients.

## RESULTS

### Prednisone increases α7 integrin levels in a mouse model and in DMD myogenic cells

**Fig. 1.**
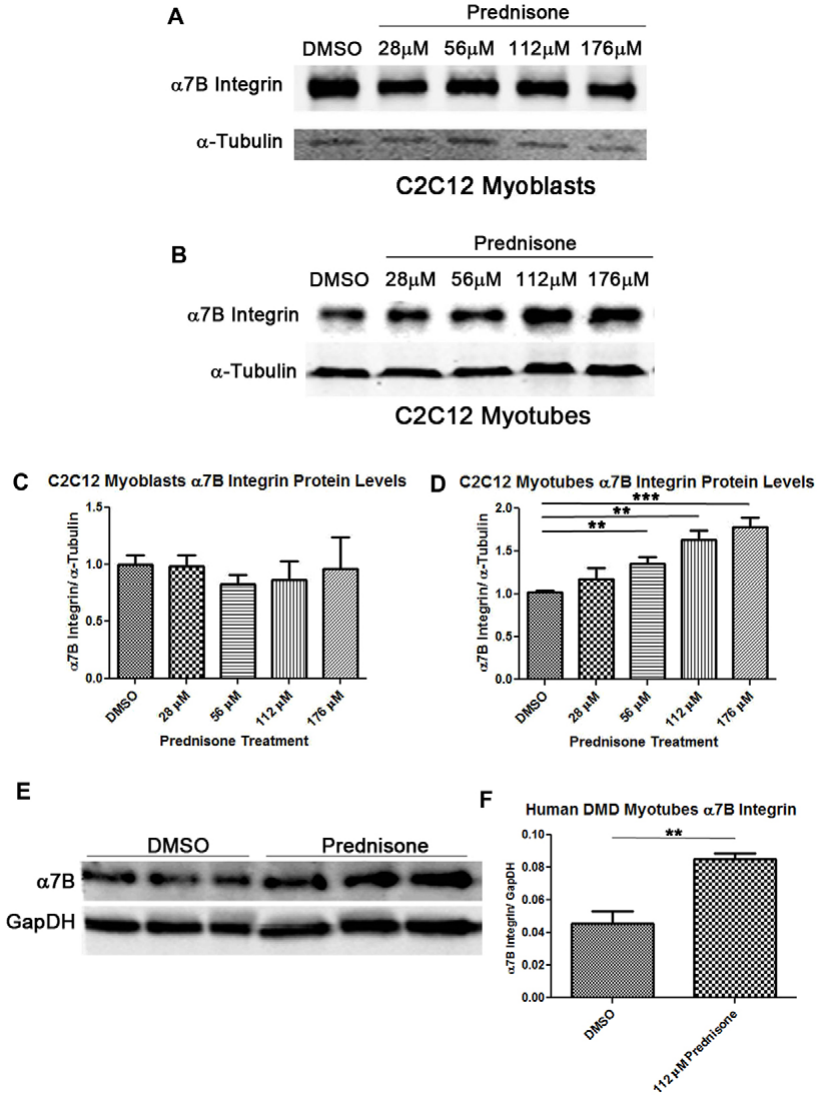
**Effects of prednisone on α7 integrin levels in C2C12 mouse myoblasts and myotubes, and human DMD myotubes.** (A,B) Western blot analysis of α7B integrin and α-tubulin from C2C12 myoblasts (A) or myotubes (B) treated with a DMSO control or increasing amounts of prednisone. (C,D) Quantitation of α7B integrin levels normalized to α-tubulin was performed and graphed for the C2C12 myoblast (C) and myotube (D) treatments (*n*=4 per treatment group, **P<0.01, ***P=0.0005). (E) Western blot analysis of α7B integrin and GapDH from cultured human DMD myotubes. (F) Western results were quantified and graphed for α7B integrin normalized to GapDH (*n*=3 per treatment group, **P=0.0021).

Corticosteroids are used to treat many chronic diseases, including DMD. In many cases, the benefits of prednisone treatment are known to occur through anti-inflammatory effects. However, inflammatory suppression does not explain the short-term benefits to muscle strength in DMD patients and the overall delay of muscle degenerative symptoms. Previous studies have shown reduced muscle pathology and improved strength in transgenic *mdx/utr*^−/−^ mice (lacking both dystrophin and utrophin) that overexpress α7B integrin (Burkin et al., 2001). We hypothesized that increased α7 integrin might be one of the mechanisms by which prednisone functions to improve muscle strength in DMD. In order to test this hypothesis, C2C12 mouse myoblasts and myotubes were treated with increasing concentrations of prednisone for 48 hours, and levels of α7B integrin protein were analyzed by western analysis and normalized to levels of α-tubulin (Fig. 1A,B). In C2C12 myoblasts, treatment with prednisone had no significant effect on α7B integrin levels compared with DMSO-treated control cells (Fig. 1A, quantified in 1C). In contrast, C2C12 myotubes showed a dose-dependent increase in α7B integrin compared with DMSO-treated control cells (Fig. 1B, quantified in 1D). Prednisone treatments of 112 μM and 176 μM resulted in a 1.6- and 1.8-fold increase in α7B integrin protein in myotubes, respectively, compared with DMSO alone. The highest dose of prednisone the cells were exposed to was 176 μM, which also gave the largest α7 integrin increase (Fig. 1D). This maximum dose was limited by both the solubility of prednisone in DMSO and cell toxicity, which was shown at >1% DMSO. These data indicate that prednisone promotes an increase in α7B integrin protein in a dose-dependent manner in cultured mouse myotubes.

Next, we examined whether prednisone treatment increased α7 integrin in human DMD myotubes. Western analysis showed that DMD myotubes treated with 112 μM prednisone had a 1.8-fold increase in α7B integrin protein compared with those treated with DMSO (Fig. 1E, quantified in 1F). These results confirm that prednisone acts to increase α7 integrin in a conserved pathway in both mouse and human myotubes.

### Prednisone increases α7 integrin in *mdx* mouse muscle

**Fig. 2.**
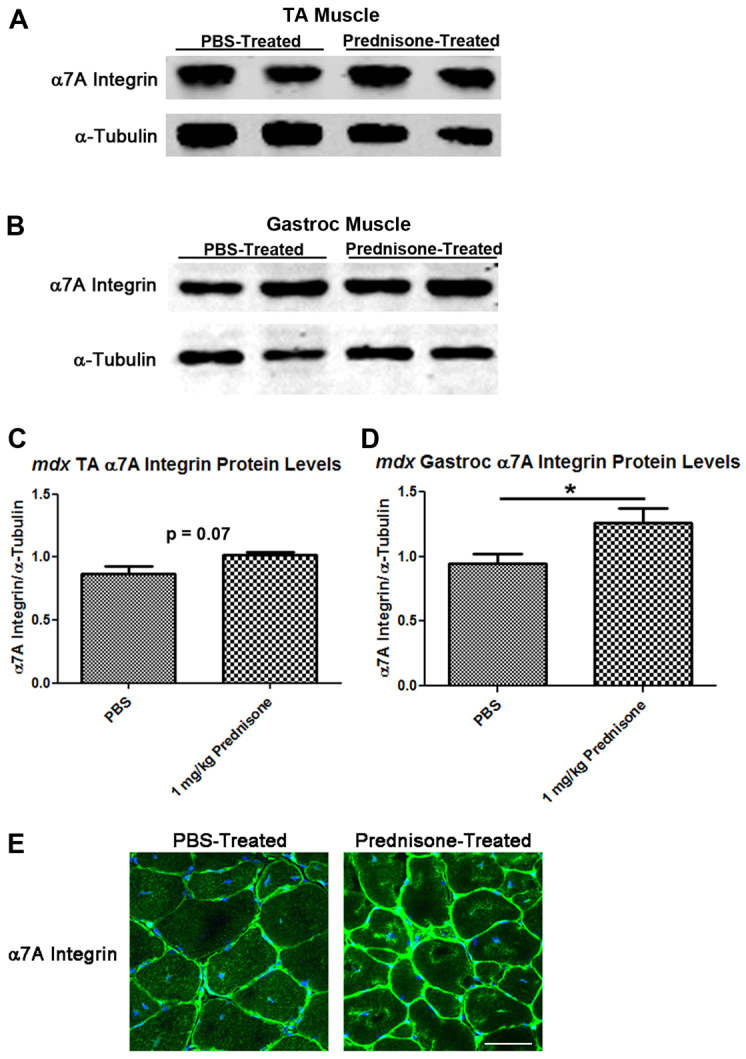
***mdx* mice treated with prednisone (1 mg/kg body weight/day) have increased α7A integrin in muscle.** (A,B) PBS-control (*n*=9) or 1 mg/kg/day prednisone (*n*=7)-treated *mdx* mouse TA (A) or gastrocnemius (B) muscle extracts analyzed for α7A integrin and α-tubulin protein levels using standard western blotting procedures. (C,D) Western blots were quantitated and graphed for α7A integrin normalized to α-tubulin for the TA (C) or gastrocnemius (*P=0.04) (D). (E) Immunofluorescence of α7A integrin from the TA of PBS-treated or 1 mg/kg/day prednisone-treated *mdx* mice. Scale bar: 50 μm.

The *mdx* mouse model for DMD was used to examine the effect of prednisone treatment on α7 integrin levels in the muscle of mice. PBS (*n*=9) or 1 mg/kg prednisone in PBS (*n*=7) was given daily by oral gavage to 3-week-old *mdx* mice. Treatment was performed for 2 weeks, at which time the mice were sacrificed and tissues harvested for analysis. We then analyzed the protein levels of α7A integrin from both the tibialis anterior (TA) and gastrocnemius muscles in prednisone-treated versus control *mdx* mice (Fig. 2A,B, respectively). In the TA we found a non-significant trend of elevated α7A integrin protein levels (∼13% increase) by western analysis (Fig. 2A); however, a significant increase (∼30% increase) was observed in prednisone-treated gastrocnemius muscles compared with controls (Fig. 2B). Results were quantified and normalized to α-tubulin (Fig. 2C,D). This differential effect is not completely surprising because previous work has shown that the TA muscle maintains lower levels of β1 integrin than the gastrocnemius muscle (Masuda et al., 2009). These results indicate that short-term treatment with prednisone increases α7A integrin in the muscle of *mdx* mice. Next, we examined the distribution of α7A integrin in the TA muscle by immunofluorescence (IF; Fig. 2E). Compared with PBS-treated mice, prednisone-treated animals showed an increase in α7A integrin at the sarcolemma, confirming western studies. These results suggest that short-term prednisone treatment within the *mdx* mouse model results in an increase in the α7A integrin protein levels at the sarcolemma.

**Fig. 3.**
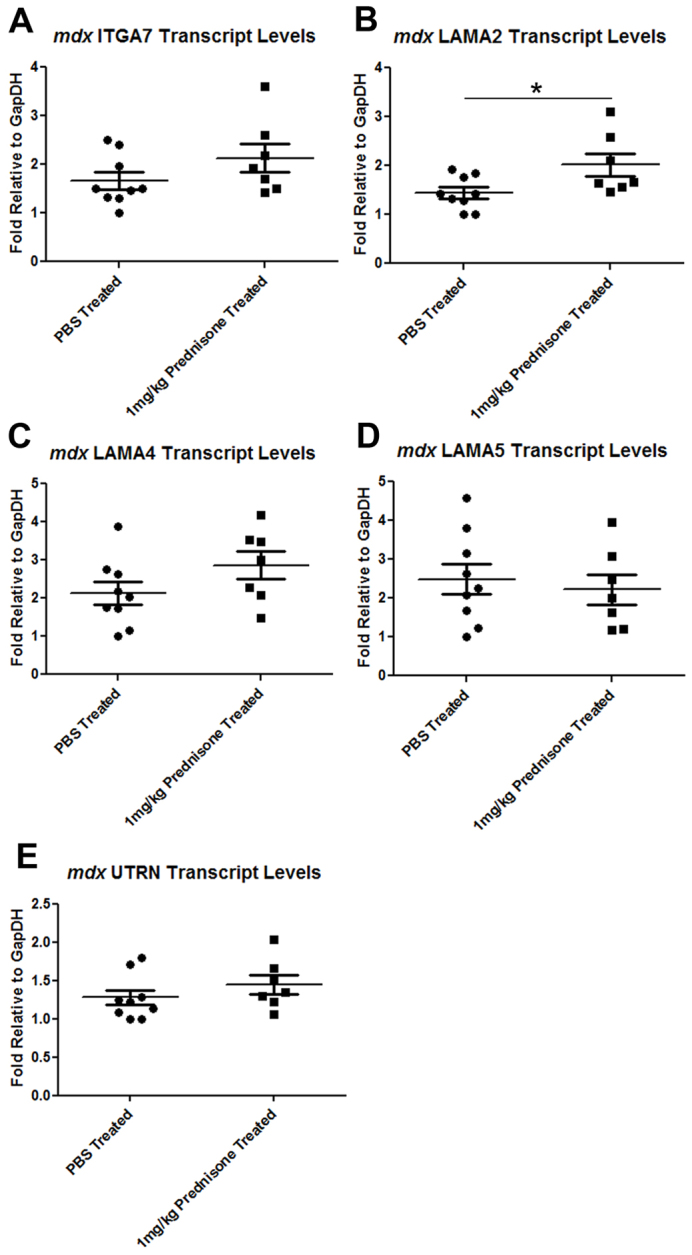
***LAMA2* transcript levels are significantly elevated in prednisone-treated *mdx* mouse muscle.** Quantitative real-time PCR was performed against *mdx* TA cDNA from animals treated with PBS (*n*=9) or 1 mg prednisone/kg body weight/day (*n*=7), using gene-specific primers against mouse *Itga7* (A), mouse *Lama2* (*P=0.03; B), mouse *Lama4* (C), mouse *Lama5* (D) and mouse *Utrn* (E).

Next, we assessed whether the prednisone-induced increase in α7A integrin protein levels in the *mdx* mouse was due to protein stabilization or increased transcription of the *Itga7* gene in muscle fibers. Quantitative real-time PCR was used to examine the transcript levels of *Itga7*, *Lama2*, *Lama4*, *Lama5* and *Utrn* relative to *GapDH* within the TA muscle of the PBS- and prednisone-treated mice (Fig. 3A–E). A 30% increase in *Itga7* transcript levels was observed with prednisone treatment, but this value did not reach significance (Fig. 3A). Similar results were obtained from triceps muscle, where prednisone treatment led to a 40% increase in *Itga7* transcript levels, albeit insignificant (supplementary material Fig. S1). These results indicate that the increased level of α7 integrin that is observed in prednisone-treated *mdx* muscle likely occurs through a transcriptionally based mechanism.

To explore the mechanism by which prednisone increased α7 integrin protein levels in skeletal muscle, we examined the transcription of laminin isoforms and utrophin in PBS- and prednisone-treated *mdx* mice. Recent studies have demonstrated that deflazacort increases laminin-α2 levels in the muscle of *mdx* mice (Anderson et al., 2000). Our results show that, compared with PBS, prednisone promoted a significant increase in *Lama2* transcripts in the TA (Fig. 3B) and triceps (supplementary material Fig. S1) muscles of *mdx* mice. There was no significant change in the levels of *Lama4*, *Lama5* or *Utrn* transcripts (Fig. 3C–E) in prednisone-treated mice. Together, these results indicate that prednisone might act to alter the laminin composition of the myomatrix and promote an increase in laminin-211 and laminin-221 in *mdx* muscle. The presence of more laminin-211 and laminin-221 in skeletal muscle basal lamina would promote stabilization of the α7β1 integrin complex in skeletal muscle, thus improving the integrity of the dystrophin-deficient sarcolemma.

### Prednisone increases α7A integrin in the muscle of GRMD dogs

**Fig. 4.**
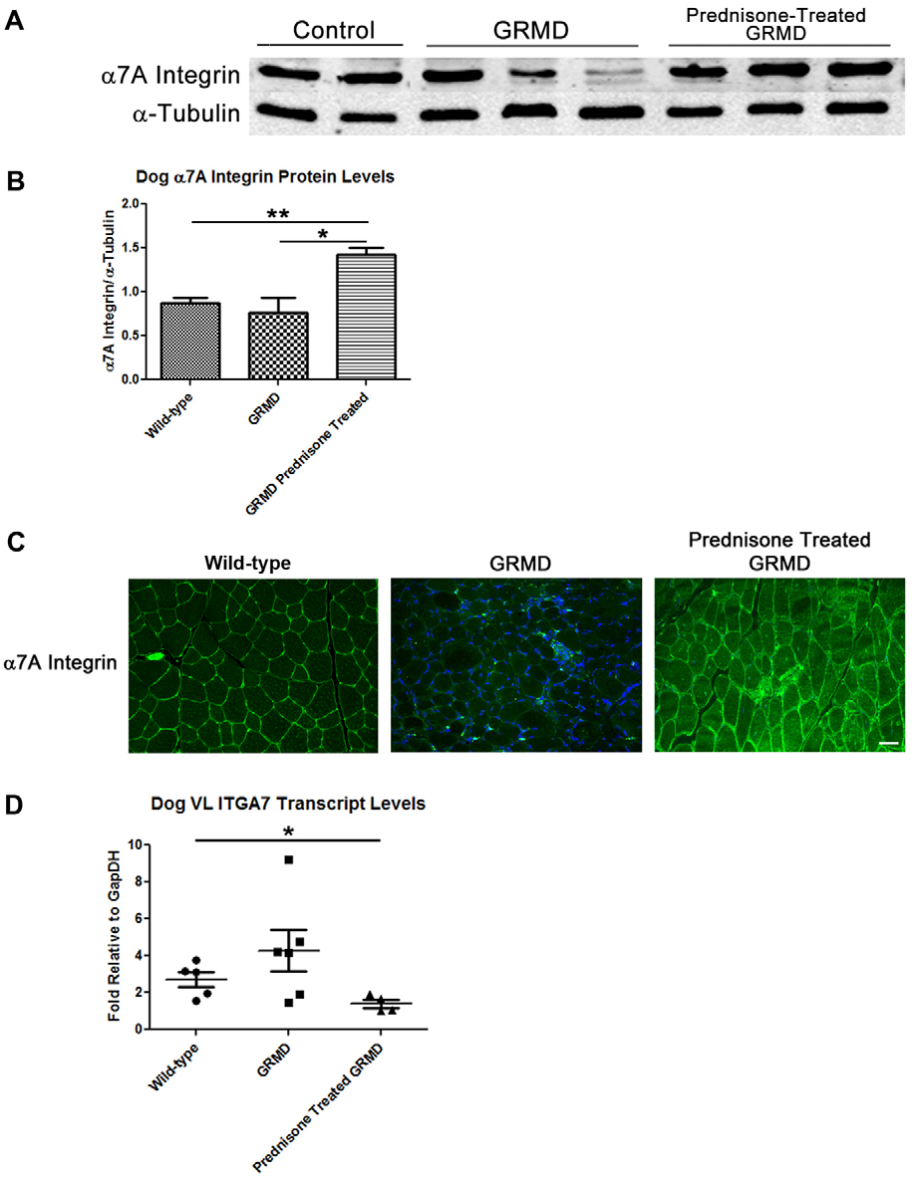
**Prednisone treatment leads to elevated α7A integrin protein and reduced *ITGA7* transcript levels in GRMD dog muscle.** (A) Representative western blot of α7A integrin and α-tubulin protein levels from the VL of control (*n*=5), untreated GRMD (*n*=6) and prednisone-treated GRMD (*n*=4) dogs. (B) Western results for α7A integrin normalized to α-tubulin were quantified and graphed (*P=0.019, **P=0.0023). (C) Immunofluorescence of α7A integrin in the VL of the three dog groups. (D) Quantitative real-time PCR of VL cDNA from wild-type (*n*=5), untreated GRMD (*n*=6) and prednisone-treated GRMD (*n*=4) dogs using primers against canine *ITGA7* (*P=0.037). Scale bar: 50 μm.

We next examined whether prednisone treatment increased α7 integrin levels in the GRMD canine model of DMD. We began by examining α7A integrin protein levels in the vastus lateralis (VL) muscle of 6-month-old wild-type, untreated GRMD dogs and prednisone-treated GRMD dogs. Using western blotting and quantitation techniques, we found a 1.7-fold increase in the levels of α7A integrin protein in the prednisone-treated GRMD dogs compared with either wild-type or untreated GRMD dogs (Fig. 4A, quantified in 4B). Although not significantly different, the average α7A integrin protein levels in untreated GRMD muscle were found to be 25% lower than that of the wild-type dogs (Fig. 4A, quantified in 4B). Furthermore, we found increased sarcolemmal-localized α7A integrin within the VL muscle of GRMD animals by immunofluorescence (Fig. 4C).

We next examined *ITGA7* transcript levels in the VL muscle of dogs using quantitative real-time PCR (Fig. 4D). Surprisingly, we found that the *ITGA7* transcript levels in prednisone-treated GRMD dogs were ∼twofold lower than untreated wild-type levels (Fig. 4D). Furthermore, although not statistically significant (*P*=0.08), the prednisone-treated GRMD dogs had average *ITGA7* transcript levels that were threefold lower compared with the levels in untreated GRMD dogs (Fig. 4D). Similar to previous findings from individuals with DMD (Hodges et al., 1997), we found a twofold increase in the average *ITGA7* transcript levels in untreated GRMD dogs compared with wild-type dogs (Fig. 4D), although again this difference was not statistically significant owing to the high variability of the transcript levels in the untreated GRMD dogs. This variability was not apparent in the *ITGA7* transcript levels of prednisone-treated GRMD dogs (Fig. 4D). Together, these results along with western data in the dog model suggest that the improved α7 integrin protein stability caused by prednisone treatment results in a negative feedback loop on *ITGA7* transcriptional activity.

### Prednisone maintains laminin-α2 protein localization and levels in GRMD dogs

**Fig. 5.**
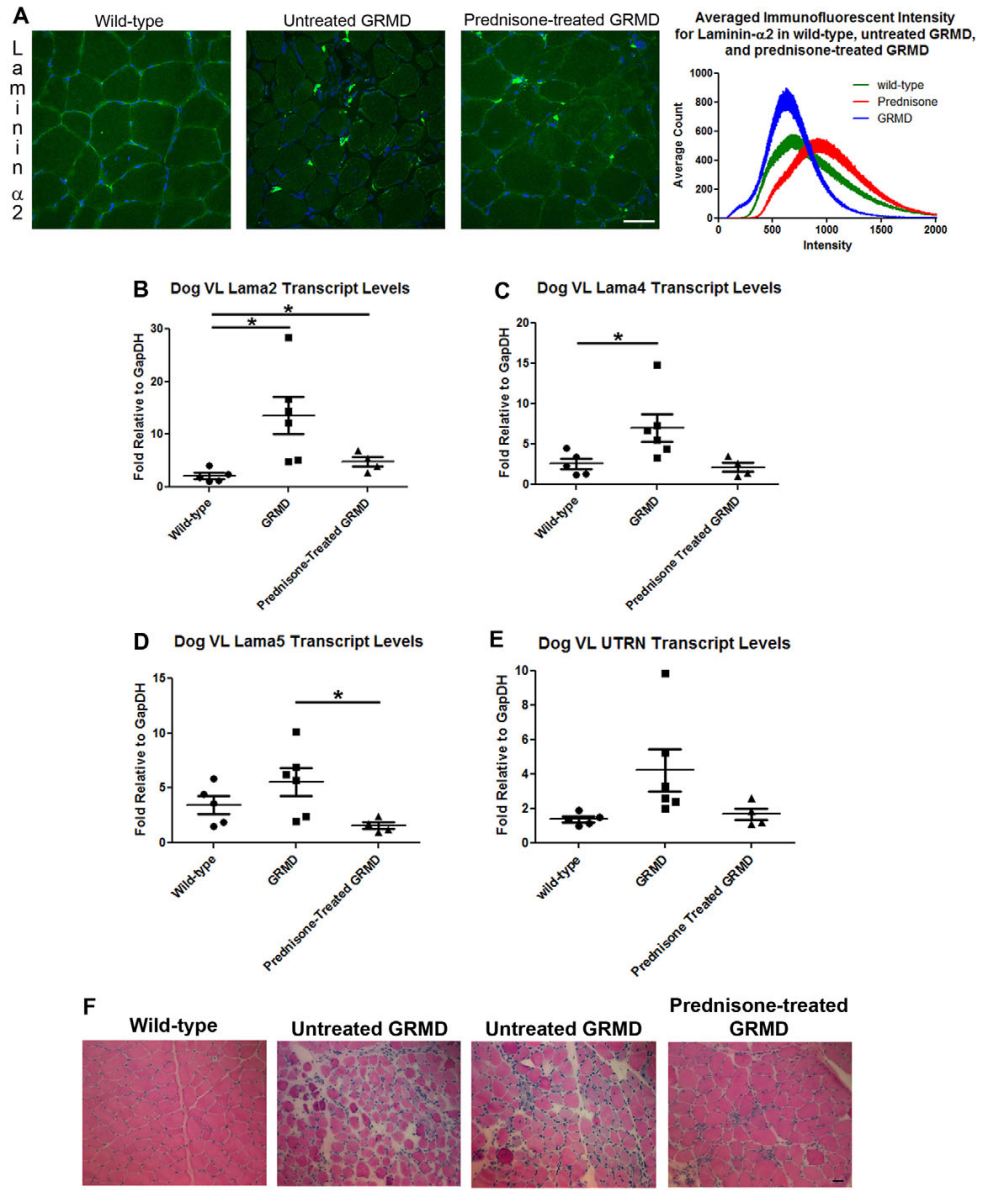
**Prednisone restores laminin-α2 protein, stabilizes transcript levels and improves muscle pathology in the GRMD dog.** (A) Immunofluorescence of laminin-α2 protein in wild-type, untreated GRMD and prednisone-treated GRMD dog VL muscle. The intensity values for two images per dog were counted, averaged by groups and plotted (see supplementary material Fig. S2). Quantitative real-time PCR of VL cDNA from wild-type control (*n*=5), untreated GRMD (*n*=6) and prednisone-treated GRMD (*n*=4) dogs using primers against canine *LAMA2* (*P<0.035) (B), canine *LAMA4* (*P=0.047) (C), canine *LAMA5* (*P=0.035) (D) and canine *UTRN* (E). (F) H&E of the VL from control, untreated GRMD and prednisone-treated GRMD dogs. The VL sections were examined and graded (Table 1) for fibrosis, inflammation and muscle fiber hypotrophy. Prednisone-treated GRMD dog tissue had less fibrosis, inflammation and fiber size disparity than the untreated GRMD dog muscle, but slightly more than that observed in wild-type dogs. Scale bar: 50 μm.

Next, we determined laminin-α2 protein levels and localization in the VL muscle of wild-type, untreated GRMD and prednisone-treated GRMD dogs using immunofluorescence. Laminin-α2 was clearly present surrounding the muscle fibers in both wild-type and prednisone-treated GRMD dogs, but was only weakly visible around untreated GRMD dog muscle fibers (Fig. 5A). Levels were semi-quantified by performing intensity measurements on images from wild-type, untreated GRMD and prednisone-treated GRMD muscle (Fig. 5A). Prednisone-treated GRMD dogs showed a 32% increase in peak relative intensity compared with wild-type muscle (Fig. 5A). Both peak intensities were higher and had different curve distributions than that observed for the untreated GRMD dog images. Untreated and prednisone-treated GRMD dogs contained numerous intense fluorescence regions of unknown origin in the muscle interstitial space that are likely to have affected measurements, especially in untreated GRMD dogs (Fig. 5A and supplementary material Fig. S2). Together, these results support that prednisone acts to increase laminin-α2 and α7 integrin protein levels in GRMD dogs.

Next, we determined whether the canine model showed differences in the transcript levels of *LAMA2*, *LAMA4*, *LAMA5* and *UTRN* (Fig. 5B–E). Like the *ITGA7* transcript levels, untreated GRMD dogs showed a large amount of individual variability in relative levels of *LAMA2*, *LAMA4*, *LAMA5* and *UTRN* transcripts, which was not observed in prednisone-treated GRMD dogs (Fig. 5B–E). *LAMA2* transcript levels were significantly increased in the VL muscle in prednisone-treated (twofold) and untreated (sixfold) GRMD dogs compared with untreated wild-type dog (Fig. 5B). The *LAMA4* (*P*=0.6), *LAMA5* (*P*=0.08) and *UTRN* (*P*=0.4) transcript levels in the prednisone-treated GRMD dogs were not significantly different compared with untreated wild-type dogs, but the *LAMA5* average was around twofold lower (Fig. 5C–E). *LAMA4* (*P*=0.051), *LAMA5* (*P*=0.03) and *UTRN* (*P*=0.13) transcript levels were around threefold lower in prednisone-treated GRMD dogs relative to the untreated GRMD dogs (Fig. 5C–E). Together, this data suggests that prednisone treatment of GRMD dogs stabilizes the transcriptional levels of all genes that we examined.

**Fig. 6.**
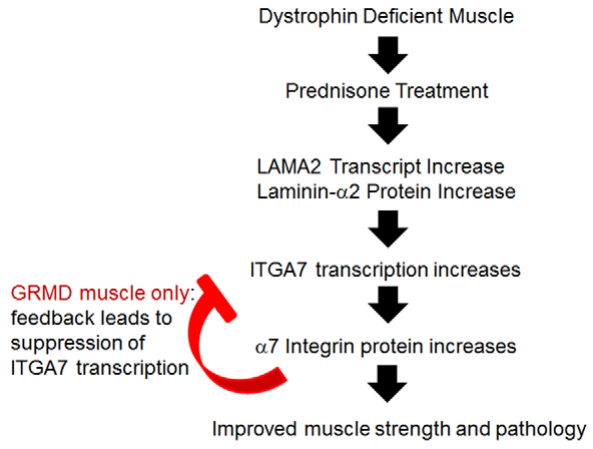
**Model depicting the action of prednisone on laminin-α2 and α7 integrin in the muscle of the *mdx* mouse and GRMD dog models of DMD.**

We were curious as to whether lesion severity would also vary between untreated and prednisone-treated GRMD dogs, and examined muscle histology by hematoxylin and eosin (H&E) staining (Fig. 5F). All GRMD dogs displayed increased myofiber size variation, fibrosis and inflammation compared with wild-type dogs. Changes in untreated GRMD dogs were more pronounced than in those treated with prednisone (Fig. 5F). Based on the overall histology, including fiber size, fibrosis and inflammation, we scored the VL sections between 1+ and 5+, with lower values suggesting greater lesion severity. These values were then summarized along with α7A integrin protein levels, laminin-α2 immunofluorescence peak intensity, and individual real-time fold-changes for *ITGA7*, *LAMA2*, *LAMA4*, *LAMA5* and *UTRN* transcripts for all dogs used in this study (Table 1). The prednisone-treated GRMD dogs had higher (more normal) lesion scores. Individual profiles from some dogs showed higher α7A integrin protein levels while exhibiting lower levels of *ITGA7* transcript and vice versa in both prednisone-treated and untreated GRMD dogs (Table 1). A similar inverse pattern was seen for the laminin-α2 protein:transcript ratio. Overall, the prednisone dose did not seem to affect protein or transcript levels in the GRMD dogs. Taken together, our data strongly suggest that a negative feedback loop exists between α7 integrin protein and *ITGA7* transcript levels in the GRMD dog model (Fig. 6). Furthermore, elevated levels of *ITGA7* and/or *LAMA2* transcripts might be indicators of a more severe muscle disease phenotype in DMD.

**Table 1.**
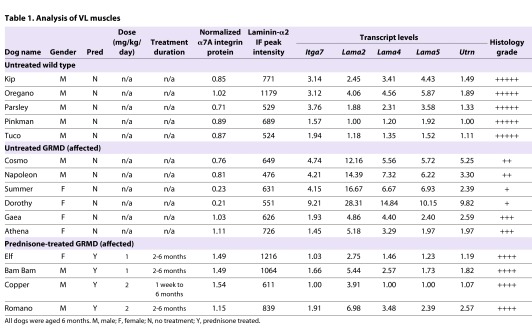
Analysis of VL muscles

## DISCUSSION

DMD is a devastating and fatal genetic disease for which there is no cure and limited treatment options. Although corticosteroids have been used for the treatment of DMD for over 20 years, our understanding of the mechanism of action of these drugs remains unclear. The therapeutic benefits of prednisone might involve a complex combination of immune modulation and strength enhancement in muscle. In this study we identified that prednisone treatment promotes an increase in the α7β1 integrin protein, a known disease modifier, in dystrophin-deficient muscle. We show that this benefit occurs in tandem with increased laminin-211/221 in the basal lamina of prednisone-treated muscle, suggesting that laminin might act to stabilize α7β1 integrin at the sarcolemma (Fig. 6). Finally, we have shown for the first time an inverse correlation between α7 integrin transcript and protein levels in the GRMD dog model for DMD. These results could profoundly alter the way we view *ITGA7* and *LAMA2* transcriptionally based data in the future.

An interesting aspect of this study was the comparison of α7 integrin levels between the *mdx* mouse and the GRMD dog. Previous work established that α7 integrin transcript levels are elevated in the muscle of *mdx* mice and in individuals with DMD (Hodges et al., 1997). The twofold increase in the *mdx* mouse α7 integrin protein levels are well defined (Hodges et al., 1997); however, the only evidence of the increase in α7 integrin protein levels from individuals with DMD is from non-quantitative immunofluorescence studies and α7 integrin transcript analysis (Hodges et al., 1997). Here we examined α7 integrin protein and transcript levels in the GRMD dog, which phenotypically resembles DMD disease progression. As with previous work from DMD patient tissue (Hodges et al., 1997), we found elevated α7 integrin gene transcripts in the untreated GRMD dog muscle compared with wild-type dogs. However, we also show that this does not correlate with an increase in α7 integrin protein. In fact, we found that an inverse correlation between the transcript and protein levels exists in the untreated GRMD dystrophic tissues. This raises the question of why this phenomenon is not present in the *mdx* mouse model, where we found a direct correlation between transcript and protein levels. GRMD dogs seem to have a negative feedback loop by which elevated α7 integrin protein levels leads to *ITGA7* transcriptional suppression. The lack of negative feedback suppression of *Itga7* transcription in the *mdx* mouse might allow for greater average α7 integrin protein levels at the sarcolemma than in other models. The mouse α7 integrin protein is more stable, lacking a secondary extracellular protease cleavage site that is conserved in rats, dogs and humans (Liu et al., 2008b). This non-cleavable mouse α7 integrin could enable the *mdx* mouse to stabilize their sarcolemma by reducing α7 integrin protein turnover, thus preventing the dystrophic progression. The severe pathology of the *mdx/α7*^−/−^ double-knockout mouse compared with the mild dystrophy in the *mdx* or *α7*^−/−^ knockout mouse lines is evidence that dystrophin and α7 integrin have overlapping roles in maintaining sarcolemmal stability in mice (Rooney et al., 2006). The severe decrease in α7 integrin protein in several GRMD dogs suggests that, in dystrophin-deficient dogs and probably humans, the α7 integrin protein is less stable than in *mdx* mice. Furthermore, the histological appearance of dystrophin-deficient dog muscle correlated with the levels of α7A integrin, suggesting that α7 integrin protein levels alone might be a major determinant of dystrophic progression. Although functional outcomes are improved subsequent to prednisone treatment in the GRMD model, histopathological changes suggest that long-term treatment could have deleterious consequences (Liu et al., 2004). Upregulation of α7β1 integrin might act to stabilize the muscle cell membrane, while independent side effects of prednisone cause pathological changes.

Because the α7B integrin protein is an important signaling molecule in muscle (Boppart et al., 2006; Liu et al., 2008a; Gurpur et al., 2009), its loss could promote a large transcriptional change through downstream signaling. Interestingly, untreated GRMD muscle, which had severely reduced α7 integrin protein levels, also showed the highest transcript levels of the genes we examined in this study. Transcript levels were comparable between prednisone-treated GRMD and wild-type dogs, with levels of *ITGA7*, *LAMA4* and *LAMA5* lower than in wild-type dogs, presumably due to the negative feedback loop. Thus, signaling feedback through elevated α7B integrin protein levels might help stabilize the transcriptional levels in the prednisone-treated GRMD dogs and greatly reduce variation in muscle transcript levels.

Increased laminin-α2 is probably part of the mechanism through which prednisone stabilizes and increases the α7 integrin protein levels at the sarcolemma. Previous data has shown that laminin-111 protein therapy increases α7 integrin in *mdx* mouse and human DMD muscle cells (Rooney et al., 2009). It is interesting that prednisone treatment led to a lower level of all transcripts observed relative to the wild-type dog except for *LAMA2* and *UTRN*. The combination of increased α7 integrin, laminin-α2 and utrophin proteins would be optimal at stabilizing the sarcolemma in the absence of dystrophin. Together, these results suggest that the elevated levels of laminin-211/221 in the myomatrix of dystrophic muscle caused by prednisone treatment might act to stabilize the α7β1 integrin at the sarcolemma, thus improving mechanical stability and strength to dystrophin-deficient muscle but also leading to altered signaling and transcriptional activity.

Studies in *mdx* mice suggest that, although short-term treatment with prednisone improves muscle strength, long-term treatment could have negative consequences on cardiac function (Sali et al., 2012). Our findings suggest that treatment with prednisone acts in part to increase laminin-α2 and α7β1 integrin protein to enhance the adhesion of skeletal muscle to the basal lamina and improve clinical outcomes. Some key questions remain concerning the action of prednisone through laminin-α2 and α7β1 integrin: (1) Why is this action of prednisone transient in dystrophin-deficient muscle? (2) Does long-term treatment with prednisone result in downregulation of the α7β1 integrin receptor and/or laminin in muscle? (3) Does prednisone have a similar mechanism of action on laminin-α2 and α7β1 integrin in the dystrophic heart? (4) Does prednisone activate known α7β1 integrin signaling pathways in muscle? (5) Does prednisone increase α7β1 integrin and laminin localization at myotendinous and neuromuscular junctions? (6) Does prednisone’s actions on laminin-α2 and α7β1 integrin extend to other types of muscular dystrophies, e.g. merosin deficient congenital muscular dystrophy type 1A.

What is clear from this study is that prednisone, a drug currently used in the treatment of DMD, acts in part through stabilization of laminin-α2 and α7β1 integrin in muscle, and our results suggest that molecules targeting or stabilizing these proteins are likely to be beneficial in the treatment of DMD.

## MATERIALS AND METHODS

### Tissue culture

C2C12 cells used in this study were purchased from ATCC and were grown in DMEM media (GIBCO, Grand Island, NY) supplemented with 20% fetal bovine serum (FBS, Atlanta Biologicals, Lawrenceville, GA), 0.5% chick-embryo extract (CEE, Seralab, West Sussex, UK), 1% L-glutamine (GIBCO, Grand Island, NY) and 1% penicillin/streptomycin (PS) (GIBCO, Grand Island, NY). C2C12 myoblasts were differentiated to myotubes in DMEM supplemented with 1% horse serum, 1% L-glutamine and 1% PS. Human DMD myoblasts were a generous gift from Dr Kathryn North (The Royal Children’s Hospital, Victoria, Australia) and used under an approved IRB from the University of Nevada, Reno. DMD myogenic cells were grown in F10 media (GIBCO, Grand Island, NY) supplemented with 20% FBS, 1% CEE, 0.5 mM CaCl_2_, 1% L-glutamine and 1% PS. Human DMD myoblasts were differentiated to myotubes in F10 media supplemented with 1% horse serum, 1% L-glutamine and 1% PS.

### Mice

The *mdx* mouse line (C57Bl10scsn-Dmdmdx) (Jackson Laboratories, Bar Harbor, ME) was used in these studies in accordance with an animal protocol approved by the University of Nevada, Reno, Institutional Animal Care and Use Committee. Mice were treated with 100 μl of PBS or 100 μl of a 200 μg/ml solution prednisone (1 mg/kg) (Sigma, St Louis, MO) by daily oral gavage for 2 weeks beginning at 3 weeks of age. At 5 weeks, mice were sacrificed and muscle tissues were surgically removed and frozen using standard procedures (Rooney et al., 2009). The TA was used for immunofluorescence, the gastrocnemius was used for western blotting, and the tricep muscle was used for quantitative real-time PCR.

### Dog tissue

All dogs were used and cared for according to principles outlined in the National Institutes of Health Guide for the Care and Use of Laboratory Animals. Archived VL muscle samples from dogs included in a prednisone preclinical trial completed at the University of Missouri-Columbia were used (Liu et al., 2004). The VL muscle was surgically biopsied at 6 months of age in five untreated wild-type dogs, six untreated GRMD dogs and four prednisone-treated GRMD dogs (two treated with 1 mg prednisone/kg body weight/day and two treated with 2 mg prednisone/kg body weight/day). GRMD prednisone treatment began at either 1 week or 2 months old and continued daily until tissue extraction (Liu et al., 2004) (see Table 1). Dogs in the 1-week treatment group were not included in the earlier published study (Liu et al., 2004).

### Western blotting

Protein was extracted from cell pellets or tissue powdered in liquid nitrogen as previously described (Rooney et al., 2009). Protein quantified using a Bradford assay and equal quantities were separated on SDS polyacrylamide gels and α7A and α7B integrin were detected as was previously described (Rooney et al., 2009). Protein loading was normalized to either α-tubulin (1:1000 mouse-monoclonal, Abcam), GapDH (1:1000, goat-polyclonal, Santa Cruz Biotechnology, Santa Cruz, CA), or the entire lane stained with Ponceau-S or Swift Stain (G-Biosciences, St Louis, MO). Quantitation was performed using ImageJ software.

### Immunofluorescence

10-μm sections of TA muscles from mice and VL muscles from dog were removed using a Leica Cryostat and immunofluorescence was performed using antibodies against α7A integrin as previously described (Song et al., 1993) or with anti-laminin-α2 antibody [1:100 goat-polyclonal, SC-16582 (C-20), Santa Cruz Biotechnology, Santa Cruz, CA] followed by FITC-donkey-anti-goat (1:1000, Jackson ImmunoResearch, Baltimore, MD) and mounted using Vectashield containing DAPI (H-1500, Vector Laboratories, CA). Images were taken using an Olympus Fluoview FV1000 Laser Confocal Microscope using consistent settings for all images, and intensity evaluation was performed using FV10-ASW3.1 software histogram function. For wild-type, untreated GRMD and prednisone-treated GRMD laminin-α2 IF analysis, two images were taken per dog section. Images were taken only from interior regions of the muscle in order to reduce any potential edge effects and to accurately reflect the overall staining of the majority of the muscle section. Intensity counts were averaged across all images for each dog group and graphed using GraphPad Prism software.

### Quantitative real-time PCR

Total RNA from powdered mouse triceps muscle and sectioned canine VL was isolated using Trizol (Invitrogen, Grand Island, NY) followed by DNase treatment (Promega, Madison, WI), and cDNA was made with random hexamers (IDTDNA) and Superscript III (Invitrogen, Grand Island, NY) using standard procedures. Quantitative real-time PCR was performed using Quanta Perfecta SYBR-Green with ROX Master Mix and was run and analyzed as previously described (Doe et al., 2011). Mouse primers for mITGA7, mGAPDH, mLAMA4, mLAMA5 and mUTRN were used as previously described (Doe et al., 2011). Mouse primers for mLAMA2 were: F- 5′-CTGGGAGTCAGCAGTCAGAAGAT-3′ and R- 5′-CTTTATGCCACTGTCCATTGCACA-3′. Primers against canine transcripts were as follows: cITGA7 F- 5′-ACTGTCCGAGCCAATATCACCGT-3′, cITGA7 R- 5′-ACCAGTAGTCCCGCCAGCACA-3′, cGapDH F- 5′-CCCCAATGTATCAGTTGTGGATCTGA-3′, cGapDH R- 5′-GGTGTCACTGTTGAAGTCACAGGA-3′, cLAMA2 F- 5′-TGGGAATCAGCAGCCAGAAAATG-3′, cLAMA2 R- 5′-GACTTTATGCCACTGTCCATCACA-3′, cLAMA4 F- 5′-GGGGAGTACCTGAATGTTCACATG-3′, cLAMA4 R- 5′-CTACATCCAACTGAACCACATTTGAATCTC-3′, cLAMA5 F- 5′-ATGAACTTCTCCTACTCGCCGCT-3′, cLAMA5 R- 5′-TAATAGTACCGGCGGGTGACGGT-3′, cUTRN F- 5′-AGCTACTCGCTGGATCCTGACCCTG-3′, cUTRN R- 5′-TGGGGCAGCAAGATGGAAATGTACT-3′.

### Statistical analysis

All statistical analysis was performed using GraphPad Prism 5 software. Averaged data are reported as the mean ± the standard error of the mean (s.e.m.). Comparison for two groups was performed using a Student’s *t*-test and between multiple groups using Kruskal-Wallis one-way ANOVA on ranks for nonparametric data. *P*<0.05 was considered statistically significant.

## Supplementary Material

Supplementary Material
