# Supplementary material for: Levels of α7 integrin and laminin-α2 are increased following prednisone treatment in the mdx mouse and GRMD dog models of Duchenne muscular dystrophy
Source: Dis Model Mech. 2013 Jul 11;6(5):1175–84. doi: 10.1242/dmm.012211 (PMC3759337; doi:10.1242/dmm.012211)
Supplement: Supplementary Material [file supp_6_5_1175__index.html]

Levels of α7 integrin and laminin-α2 are increased following prednisone treatment in the mdx mouse and GRMD dog models of Duchenne muscular dystrophy — Levels of α7 integrin and laminin-α2 are increased following prednisone treatment in the mdx mouse and GRMD dog models of Duchenne muscular dystrophy — Supplementary Material 

# Levels of α7 integrin and laminin-α2 are increased following prednisone treatment in the *mdx* mouse and GRMD dog models of Duchenne muscular dystrophy

## DMM012211 Supplementary Material

**Files in this Data Supplement:**

- **Supplementary Material PDF**
