# Supplementary material for: Levels of α7 integrin and laminin-α2 are increased following prednisone treatment in the mdx mouse and GRMD dog models of Duchenne muscular dystrophy
Source: Dis Model Mech. 2013 Jul 11;6(5):1175–84. doi: 10.1242/dmm.012211 (PMC3759337; doi:10.1242/dmm.012211)
Supplement: Supplementary Material [file supp_012211_DMM012211.pdf]

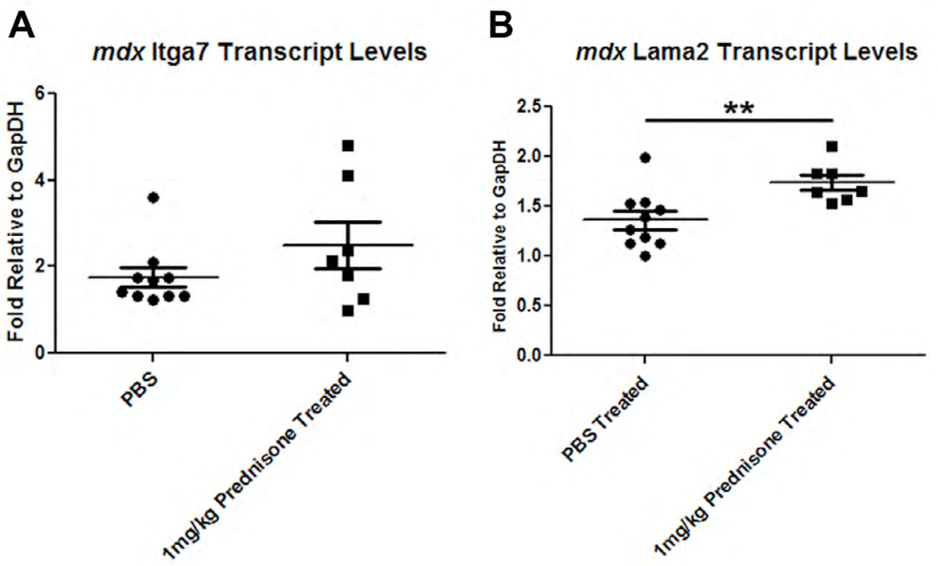

Fig. S1. Quantitative real-time PCR of mouse *Itga7* (A) and mouse *Lama2* (***P*=0.001) (B) from mdx triceps muscle.

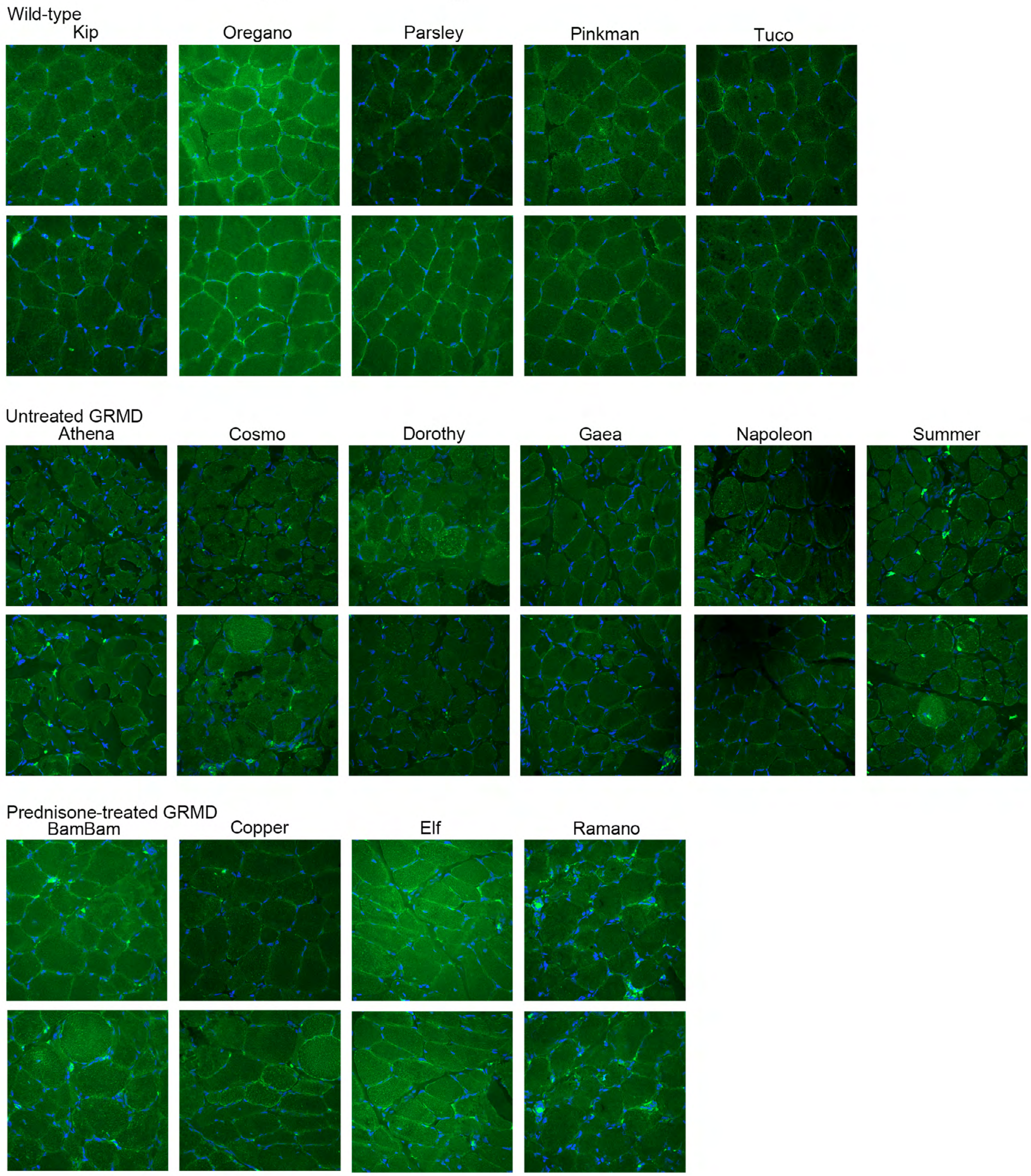

Fig. S2. Immunofluorescence images from individual dogs which were used for fluorescent quantification. Images are displayed under the individual dogs name and have been grouped into wild-type, untreated GRMD, and prednisone-treated GRMD in order to better observe similarities and differences between individuals and groups. (Scale bar=50 μm).
